# Evaluating the efficacy of the praziquantel dose pole for schistosomiasis treatment: A multi-country systematic review

**DOI:** 10.1371/journal.pntd.0013587

**Published:** 2025-10-10

**Authors:** Paprika Berry, Pedro H. Gazzinelli-Guimaraes

**Affiliations:** Department of Microbiology, Immunology and Tropical Medicine, School of Medicine and Health Sciences, George Washington University, Washington, DC, United States of America; George Washington University School of Medicine and Health Sciences, UNITED STATES OF AMERICA

## Abstract

Schistosomiasis is a neglected tropical disease targeted for elimination as a public health problem by 2030 under the WHO’s roadmap. Praziquantel, administered via the dose pole, which estimates the number of tablets needed for treatment according to an individual’s height, has been used as preventive chemotherapy (PC) for 25 years, particularly for school-aged children (SAC). In 2022, PC was required for schistosomiasis in 50 countries, affecting a total of 264.3 million people, including 129.4 million adults. This systematic review evaluates the accuracy of the praziquantel dose pole across 64,212 individuals from 21 countries, focusing on its efficacy in delivering optimal, acceptable, insufficient, and excessive doses during school-based and community-wide treatment campaigns for schistosomiasis. The search strategy included the terms “dose pole” OR “tablet pole” AND “praziquantel” AND “schistosomiasis” in four databases (PubMed, Scopus, LILACS, and Embase), ranging from 1999 to September 2024. The dose pole demonstrated 96% accuracy in providing optimal/acceptable doses (30–60mg/Kg) to SAC. However, its effectiveness markedly declined for adults (15–95 years), with a pooled proportion of 19% receiving insufficient doses (<30mg/Kg), ranging from 10% to 34%. These discrepancies arise from misalignments between adult body metrics and the dose pole’s height-based dosing, primarily due to overweight and obesity, leading to underdosing. While the dose pole remains effective for SAC, its limitations for adults necessitate urgent adaptation or alternative dosing strategies to ensure equitable and effective treatment across all age groups.

## Author summary

Human schistosomiasis is a parasitic disease caused by trematodes of the genus *Schistosoma,* which is transmitted through exposure to contaminated water and is endemic in 78 countries. The World Health Organization has created a road map for the elimination of schistosomiasis as a public health problem by 2030. Treatment is provided in endemic areas in the form of praziquantel preventive chemotherapy, given during mass drug administration campaigns using the dose pole. The dose pole estimates the dosage of praziquantel tablets based on a person’s height rather than weight and is employed in both school-based and community-wide treatment programs. This review highlights the inaccuracies of the dose pole to provide an acceptable/optimal dose of praziquantel for treatment, especially for those where height and weight misalign, most commonly found with those with higher BMIs, adults, and adolescent girls. To address these underdosing issues, modifying the dose pole is suggested; without improvements, dosing inaccuracies will continue in these endemic areas, hindering efforts to interrupt transmission.

## Introduction

Human schistosomiasis is a parasitic disease caused by several species of the blood fluke trematode from the genus *Schistosoma*, etiological agents of two clinical forms: intestinal schistosomiasis (caused by *Schistosoma mansoni, S. japonicum*, *S. mekongi, S. intercalatum, and S. guineensis*) and urogenital schistosomiasis (caused by *S. haematobium)*. Infections result from exposure to contaminated water with cercaria that penetrate human skin after being released by snails, the intermediate host [1]. Found globally in tropical areas, schistosomiasis is endemic to specific regions in South America, Asia, and Africa, with over 90% of the global burden of schistosomiasis concentrated in sub-Saharan Africa [2,3]. The World Health Organization (WHO) has designated schistosomiasis as one of the top neglected tropical diseases (NTDs) and has prepared a roadmap targeting its elimination as a public health problem by 2030 [3,4]. Schistosomiasis infections contribute to morbidity with clinical manifestations of anemia, diarrhea, abdominal pain, blood in urine, and stunting. In 2022, globally, schistosomiasis accounted for 1.4–3.3 million disability-adjusted life years (DALYs) [3]. Infected but untreated people in endemic areas often develop chronic infections with clinical manifestations due to egg accumulation and immunological responses to antigens, characterized by liver or bladder inflammation and fibrosis [5]. Also, in women, urogenital schistosomiasis infections have been implicated as a risk factor for Human Immunodeficiency Virus (HIV) [6]. Control methods for schistosomiasis typically focus on interrupting the parasite’s life cycle and, consequently, transmission, including large-scale preventive chemotherapy (PC) with praziquantel, behavioral changes, vector control (molluscicides), and improved Water, Sanitation, and Hygiene (WASH) [3]. Praziquantel is a low-cost, effective drug for treating schistosomiasis. Praziquantel is used as preventative chemotherapy, typically administered as a single dose during mass drug administration campaigns (MDA) in endemic areas and administered by a dose pole. Praziquantel has been extensively and successfully used for the past 40 years; it is administered based on the recipient’s weight, optimally at 40 mg/Kg when treating *S. mansoni* and *S. haematobium* [7]. However, most studies evaluating treatment outcomes for schistosomiasis have relied on a single 40 mg/kg dose, reporting cure rates ranging from 39.8% to 88.9%, predominantly in school-aged children years old years old [8–10]. In contrast, multiple-dose regimens have achieved substantially higher cure rates, ranging from 53.1% to 100.0% [11,12], including a recent trial that reported a 96.4% cure rate following the monthly administration of 60 mg/kg for three consecutive months [13]. In addition, praziquantel’s exclusive large-scale use calls into question the possibility of resistance and increases the need for the development of alternative drugs [7,14,15].

In 2022, according to the WHO, 89.1 million individuals (68.6 million school-aged children and 20.5 million adults) received PC for schistosomiasis using praziquantel via mass drug administration (MDA) programs. For regions where the prevalence of Schistosoma spp. infection is 10% or higher, the latest WHO guidelines recommend administering annual PC—a single dose of praziquantel—with at least 75% coverage among all individuals aged 2 and above, including adults, pregnant women beyond the first trimester, and lactating women. Moreover, in the lack of appropriate response to MDA even when 75% treatment coverage is achieved (hotspots areas), WHO advises that biannual rather than annual MDA should be considered. The dose pole is a six-foot-long pole with six intervals, which estimates the number of tablets needed for treatment according to an individual’s height calculations [16–21]. Initially, the dose pole was designed to treat school-age children, with five intervals (1.5, 2, 2.5, 3, 4 tablets). The height ranges from 110 to 178 cm, corresponding to doses related to the child’s height. Hall and collaborators [16] tested this pole design, which identified height as the best measurement for estimating weight in children. Following the development of the dose pole, Montresor et al. [19] set out to validate the pole by reviewing data from over 25,000 children from 10 countries, in more than 98% of the cases doses fall within the range that has historically been deemed safe (30 and 60 mg/kg), with only 2% of those children not falling into the height ranges of the pole [19]. This range of 30–60 mg/kg has been used in the field as a systematic approach to dosing praziquantel; however, new controlled and field studies are needed to validate this range. Following this, in 2005, the WHO dose pole was successfully modified by adding two intervals between 94 cm and 110 cm (corresponding to 1 tablet) and over 178 cm (5 tablets), with only 1% of children being underdosed, which became the current WHO dose pole. This dose pole was tested on height/weight data from 9,356 individuals (predominantly children) from 11 non-African countries, with 98% of the individuals receiving an acceptable dosage (30–60 mg/kg) [17].

In 2010, although not incorporated into the current version, a proposal suggested adding two intervals (1/2 and 3/4) to extend the pole to include shorter heights (60–99 cm), thereby treating preschool-aged children and infants [18]. Indeed, praziquantel has been suggested for this age range; however, limitations have arisen due to the need to divide a standard praziquantel tablet, which can lead to dosing errors and a deterrent taste [22]. The formulation of a new pediatric arPraziquantel tablet (L-praziquantel) for preschool children (3 months to 6 years) has also been used to bridge this treatment gap, showing a cure rate similar to that observed with the standard praziquantel [22]. Therefore, an argument has been made for developing a universal pole for infants and children.

Moreover, the updated WHO guidelines [3,4] have significantly expanded the age range of individuals targeted for the schistosomiasis elimination program, including a substantial number of adults eligible for treatment. In 2022, 129.4 million adults required a PC. This modification has shifted the approach of PC from a treatment predominantly based in schools to comprehensive community-wide treatment in the endemic areas for schistosomiasis. Therefore, the efficiency of the current format of the praziquantel dose pole in delivering acceptable or optimal doses of praziquantel to adults needs to be tested and validated in a multi-country approach. Over the last decade, several isolated studies have reported a significant number of underdosing of praziquantel administered by the dose pole in adult populations in Africa [21,23,24].

With this systematic review, we sought to answer the question: “What is the accuracy of the current praziquantel dose pole in treating children and adult populations for schistosomiasis in endemic areas?”

## Methods

### Search strategy and selection criteria

Using the Preferred Reporting Items for Systematic Reviews and Meta-Analyses extension [25], we searched for papers that explored the performance of the dose pole to administer accurate praziquantel doses to treat schistosomiasis in settings that included school-based SAC, children (including those outside of school), and community-based treatment programs (children and adults) in different endemic regions worldwide. Papers were retrieved from four databases: U.S. National Institutes of Health’s Library (PubMed), Scopus, LILACS, and Embase, with the search period from 1999 to September 2024. The keyword strings “dose pole” OR “tablet pole” AND “praziquantel” AND “schistosomiasis” were used to conduct the searches.

The inclusion criteria for this review consisted of identifying acceptable (30–40 mg/kg), optimal (40 mg/kg), insufficient (<30 mg/kg), and excessive (>60 mg/kg) dosages, in line with the WHO categorizations. School-based treatment studies specifically mentioned the use of the pole in a school setting and the use of the dose pole in school-aged children. In papers where Community-wide treatment was identified, the dose pole was used to administer praziquantel outside of schools to reach children who do not attend school, as well as potentially high-risk adults. Papers included in this review also had to compare the delivery of praziquantel using the dose poles predicted tablets and the dose based on body weight calculation for schistosomiasis treatment.

Following the return of the search queries, results from all the databases were compared, and duplicate papers were removed. Initially, the resulting papers were reviewed by two investigators independently to ensure that the selected papers met the inclusion criteria. When these papers were reviewed, if available, abstracts and full-text were evaluated in parallel by both reviewers and were compared between reviewers for inclusion. The inclusion criteria for this review were defined as the comparison between the dose the person received based on the dose pole and their actual dose based on body weight, as measured using a scale. Papers were grouped according to the method of drug administration programs/methods: school-based treatment with children, community-wide treatment with SAC and adults, and community-wide treatment with adults only. In this review, some papers using a school-based treatment approach included people from 1 to 22 years old. We recognize, as defined by WHO, that typically children are 1–15 years old. Then, the data were further split into subgroups to include these individuals aged 15 years or older as adults in age-matched evaluations (15–23 and 15–45). This method of stratifying corresponded with the criteria used for inclusion in this review.

Data were collected from each original paper, typically from tables generated by the authors, and when raw data were available, numbers were tabulated again. This data included the number of people treated in each dosage range, their ages, sex (if available), dosage accuracy (optimal or less than optimal received), and the type of treatment (community or school-based). New tables were produced based on the treatment methods and totals for the treatment program, categorized by each dosage, and analyzed and verified by the two investigators. When dosage groups and raw data did not explicitly categorize data were available, the data were reevaluated for classification. Precise age range data were missing for one study; nevertheless, the paper directly states that the study was conducted in the target group (SAC). Based on the data retrieved from the authors’ papers, 95% confidence intervals (CI) were calculated for the overall totals in each table.

Notably, in Hall et al. [16], the dose ranges were classified differently from the WHO classifications. Insufficient dose was considered <36 mg/Kg in this study, and the optimal dose was considered as 36–44 mg/Kg; these were in a similar range, so the paper was accepted. Another paper identified the age range as younger than what would be considered school-aged; however, the ages were in a population of children eligible for treatment with the dose pole and were therefore included [26].

The exclusion criteria included papers identified as reviews, abstracts, notes, clinical trials, and agendas. Specifically, Strandgaard et al. [27] conducted a clinical trial that focused primarily on dose pole usage to treat opisthorchiasis with praziquantel. In addition, some studies mentioned the use of the dose pole for treatment but did not mention the use of body weight calculations for dose comparison and were excluded [28,29] (S1 Dataset).

### Praziquantel dose range

The WHO has defined the ideal standard of praziquantel treatment for schistosomiasis as 40 mg/kg, but the optimal dose is 40–60 mg/kg. However, an acceptable dose has been defined as 30–40 mg/kg. Some doses fall outside these ranges, with the dose <30 mg/kg defined as insufficient and >60 mg/kg as excessive. For inclusion in this review, papers must compare administered dosages using a dose pole and calculate the receiver’s body weight.

### Statistical meta-analysis

We performed a statistical analysis to quantitatively synthesize the proportion of individuals receiving optimal, acceptable, insufficient, or excessive praziquantel doses, using the WHO dose pole in both school-based treatment and community-wide treatment settings. Proportions and 95% confidence intervals (CIs) were calculated using the Wilson score method (S2 Dataset). For the meta-analysis, we applied a DerSimonian and Laird random-effects model with logit transformation to stabilize variances and account for between-study heterogeneity. The between-study variance (τ²) was estimated from the Q statistic and used to calculate inverse-variance weights. Back-transformation of pooled logit estimates provided an overall proportion and 95% CI of individuals receiving insufficient, optimal, acceptable, or excessive dosing. Forest plots were generated to visualize individual study estimates and the pooled proportions. All analyses were conducted in Python using custom scripts with the statsmodels and matplotlib libraries.

## Results

**Fig 1:**
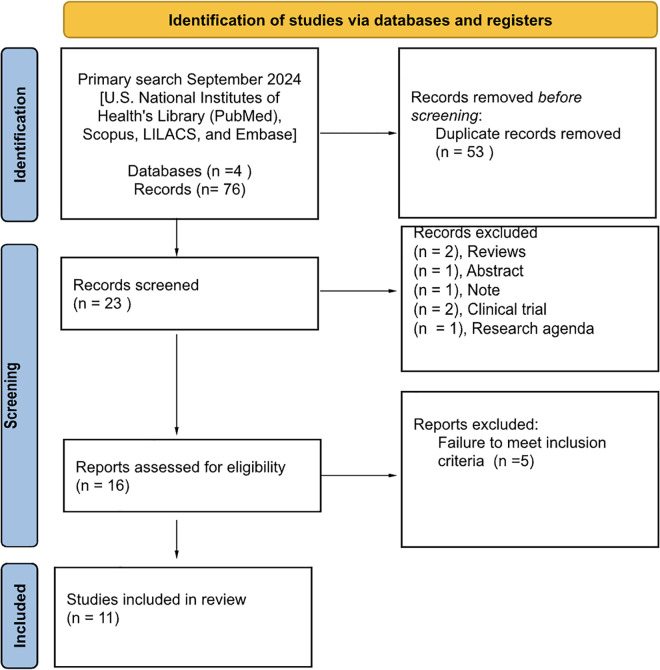
PRISMA Flow diagram. Diagram outlining the identification of papers through a search conducted in September 2024, results retrieved from four databases with screening and review of studies, and those subsequently acquired for the systematic review.

In this systematic review, 76 papers, published between 1999 and September 2024, retrieved from four different databases (U.S. National Institutes of Health’s Library (PubMed), Scopus, LILACS, and Embase) were identified, using the keywords “dose pole” or “tablet pole” and “praziquantel” and “schistosomiasis”. From this search, 53 duplicates were removed, and 23 papers were selected for screening. Following, seven records were excluded due to the nature of the paper: reviews (n = 2), abstracts (n = 1), notes (n = 1), clinical trials (n = 2), and research agendas (n = 1). The remaining 16 reports were screened for inclusion eligibility, and five were further excluded due to failure to meet the inclusion criteria (S1 Dataset). Therefore, 11 studies were included in this systematic review, comprising 64,212 people (aged 0–95 years old) from 21 endemic countries for schistosomiasis, eight non-African countries, and 13 African countries (Fig 1).

Nine studies were identified as treating school-aged children in school-based treatment campaigns [16–21,24,30,31]. Six studies were identified as treating both adults and children within the community-wide treatment approach [17,19,21,23,24,26], and three papers were classified as community-wide treatment for adults only [21,23,24].

The current WHO dose pole was specifically designed to treat SAC and tested with thousands of children to determine the optimal dosage intervals [16,17,19,20]. Firstly, this review focused on dose pole usage in that target demographic. From nine studies, 45,950 students from 17 countries, aged between 0 and 22, received treatment using the dose pole in a school-based program. From this group, over 93% of the children received an adequate dose (30–60mg/Kg), with 75% ([75.3%-75.5%]) (34,649) children receiving an optimal dose, and 18.1% ([18.01-18.19%]) (8,328) receiving an acceptable dose (Table 1). Some received less than acceptable doses of praziquantel, with 3% (1,381 SAC) receiving an insufficient dose and 3.5% (1,592) receiving an excessive dose.

**Table 1:** School-based treatment (ages 0-22) with praziquantel using the WHO dose pole for treatment based on classifications (Insufficient, Acceptable, Optimal, and Excessive doses) from nine studies.

School-based treatment							
Country	Sample Size	Age range	Insufficient dose <30 mg/Kg	Acceptable dose 30-40 mg/Kg	Optimal dose 40-60 mg/Kg	Excessive dose >60 mg/Kg	Reference
3 African countries^a^	5,950	6-17	635 (10.67%)*	4,455(74.87%)**		860 (14.45%)	Hall et al., 1999 [10]
7 African countries^b^	19,923	1-20	99 (0.5%)	2,765 (13.8%)	16,884 (84.8%)	175 (0.9%)	Montresor et al., 2001 [18]
Tanzania	1,287	N/A	6 (0.5%)	273 (21.2%)	1,004 (78%)	4 (0.3%)	Montresor et al., 2002 [19]
Tanzania	1,477	5-18	4 (0.3%)	420 (28.4%)	1,047 (70.9%)	6 (0.4%)	Nordin et al., 2014 [30]
6 non-African countries^c^	3,979	7-18	63 (1.6%)	707 (17.8%)	3,178 (79.8%)	31 (0.8%)	Montressor et al., 2005 [11]
South Africa	1,008	10-12	108 (10.7%)	510 (50.6%)	389 (38.6%)	1 (0.1%)	Baan et al., 2016 [9]
Uganda	3,303	0-6***	7 (0.2%)	746 (22.6%)	2,203 (66.7%)	347 (10.5%)	Sousa-Figueredo et al., 2010 [12]
Philippines	1,427	8-22	3 (0.2%)	89 (6.23%)	1,334 (93.5%)	1 (0.07%)	Erfe et al., 2013 [21]
Mozambique	7,596	5-15	456 (6%)	2,818(37.1%)	4,155 (54.7%)	167 (2.2%)	Gazzinelli-Guimaraes et al., 2018 [22]
**Total**	**45,950**	**0-22**	**1,381** **(3% [±0.03])**	**8,328** **(18.1% [±0.09])**	**34,649** **(75.4% [±0.1])**	**1,592** **(3.5% [±0.03])**	

a.Ghana, Malawi, and Tanzania. b. Chad, Ghana, Guinea, Kenya, Mali, Mozambique, Tanzania. c. Brazil, China, Indonesia, Malaysia, Oman, Yemen. d. Madagascar, Mali, Senegal, Tanzania, Uganda. * Insufficient dose considered <36 mg/Kg in this study. **The optimal dose considered in this study was 36–44 mg/Kg. *** Current WHO dose pole tested for preschool-aged children.

**Fig 2:**
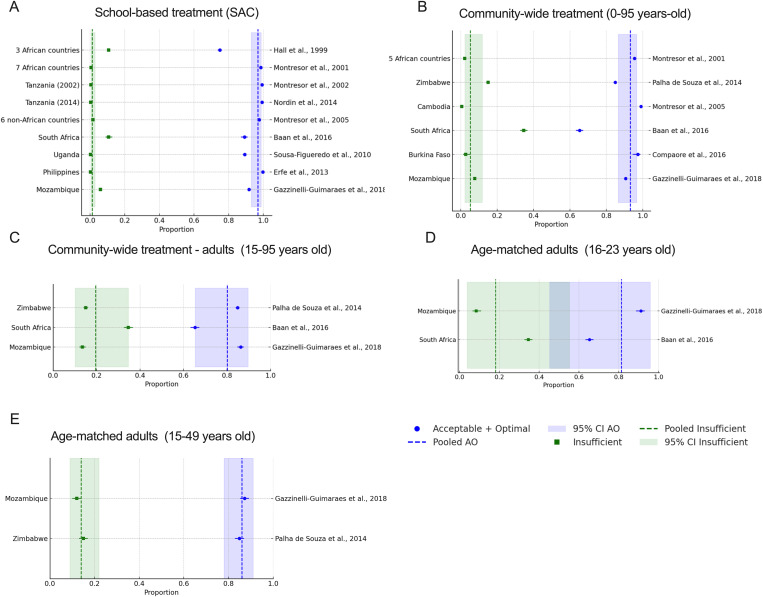
Random-effects meta-analysis of acceptable/optimal and insufficient praziquantel dosing by treatment strategy and age range. Forest plots show the proportion of participants receiving acceptable/optimal doses (30-60 mg/Kg) (blue circles) and insufficient doses (<30mg/Kg) (green squares) for each study, estimated using the DerSimonian–Laird random-effects model. Panels represent: (A) school-based treatment of school-age children (SAC), (B) community-wide treatment including SAC and adults (0–95 years), (C) community-wide treatment of adults only (15–95 years), (D) age-matched adults (16–23 years), and (E) age-matched adults (15–49 years). The dotted vertical line indicates the pooled proportion for each dosing category, and the shaded area shows the corresponding 95% confidence interval (CI).

Interestingly, random-effects meta-analysis of these nine studies from the school-based treatment setting showed a pooled estimate of 96.8% (95% CI: 92.9% to 98.6%) of children receiving an acceptable or optimal dose using the WHO dose pole (S3 Dataset and Fig 2), with only 1.3% (95% CI: 0.6% to 2.8%) of insufficient dose. These findings confirm the high accuracy and reliability of the current dose pole in school-aged children across diverse studies and support its continued use in school-based mass drug administration programs.

The community-wide treatment setting, including SAC and adults, utilized data from six studies involving 25,858 individuals, aged 0–95, from ten countries. Overall, in this group, 50.2% [50.03-50.37%] (12,992) of the people received an optimal dose, and 39% [38.90-39.10%] (10,086) received an acceptable dose (Table 2). However, the meta-analysis revealed that the frequency of insufficient doses increased from 1.3% in SBT to 5.4% [2.3-11.9%] in CWT, considering both SAC and adults. Notably, we calculated the performance of the dose pole for schistosomiasis in the adult population only during community-wide treatment, based on data from three studies. The analysis of 9,994 adults, ages 15–95 in Mozambique, South Africa, and Zimbabwe, demonstrated that 52.5% [52.49-52.5%] (5,248) received an acceptable dose, and only 28.5% [28.4-28.6%] (2,846) received an optimal dose. In addition, a marked 18.8% [18.69-18.91%] (1,887) received an insufficient dose, and 0.1% [13] received an excessive dose (Table 3).

**Table 2:** Community-wide treatment (ages 0-95) classified based on the dose categories (Insufficient, Acceptable, Optimal, and Excessive doses), from six studies.

Community-wide treatment							
Country	Sample Size	Age range	Insufficient dose <30 mg/Kg	Acceptable dose 30-40 mg/Kg	Optimal dose 40-60 mg/Kg	Excessive dose >60 mg/Kg	Reference
5 African countries^d^	4,710	1-83	103 (2.1%)	1,284 (27.3%)	3,210 (68.2%)	113 (2.4%)	Montresor et al., 2001 [18]
Zimbabwe	5,614	15-49	842 (15.0%)	2,970 (52.9%)	1,796 (32.0%)	6 (0.1%)	Palha de Souza et al., 2014 [23]
Cambodia	3,300	1-64	18 (0.6%)	670 (20.3%)	2,598 (78.7%)	14 (0.4%)	Montressor et al., 2005 [11]
South Africa	2,149	16-23	744 (34.6%)	1,104 (51.4%)	299 (13.9%)	2 (0.1%)	Baan et al., 2016 [9]
Burkina Faso	258	N/A****	7 (2.7%)	131 (50.8%)	120 (46.5%)	0 (0%)	Compaore et al., 2016 [14]
Mozambique	9,827	5-95	757 (7.7%)	3,927 (39.96%)	4,969 (50.56%)	174 (1.77%)	Gazzinelli-Guimaraes et al., 2018 [22]
**Total**	**25,858**	**0-95**	**2,471** **(9.6% [±0.09])**	**10,086** **(39% [±0.10])**	**12,992** **(50.20% [±0.17])**	**309** **(1.20% [±0.008])**	

d. Madagascar, Mali, Senegal, Tanzania, Uganda. **** Age range not reported; only all ages from two genders were assessed.

**Table 3:** Community-based treatment for adults (ages 15-95) by dose categories (Insufficient, Acceptable, Optimal, and Excessive) using the WHO dose pole, from three studies in Zimbabwe, South Africa, and Mozambique.

Community-based treatment (Adults only)							
Country	Sample Size	Age range	Insufficient dose <30 mg/Kg	Acceptable dose 30-40 mg/Kg	Optimal dose 40-60 mg/Kg	Excessive dose >60 mg/Kg	Reference
Zimbabwe	5,614	15-49	842 (15.0%)	2,970 (52.9%)	1,796 (32.0%)	6 (0.1%)	Palha de Souza et al., 2014 [23]
South Africa	2,149	16-23	744 (34.6%)	1,104 (51.4%)	299 (13.9%)	2 (0.1%)	Baan et al., 2016 [9]
Mozambique	2,231	16-95	301(13.5%)	1,174 (52.6%)	751 (33.7%)	5(0.2%)	Gazzinelli-Guimaraes et al., 2018 [22]
**Total**	**9,994**	**15-95**	**1,887** **(18.8% [±0.11%])**	**5,248** **(52.5% [±0.007%])**	**2,846** **(28.5% [±0.10%])**	**13** **(0.1%** **[±0.0005%])**	

The random-effects meta-analysis for community-wide treatment, including only adults (ages 15–95), estimated that 80.1% (95% CI: 65.3%-89.6%) received acceptable or optimal doses. However, we observed a marked decline in the frequency of optimal dosing (40–60 mg/kg) when comparing SAC from school-based treatment [73.9% (95% CI: 63.4%-82.2%)] to the adult population [25.3% (95% CI: 16.1%-37.4%)]) in CWT using the WHO dose pole (S1 Fig and S4 and S5 Datasets). In parallel, there was a substantial increase in the proportion of adults receiving insufficient doses (<30 mg/kg) at 19.6% (95% CI: 10.1%-34.5%) from 1.3% in SAC (Fig 2 and S3 Dataset), corresponding to a significant odds ratio of 18.20 (95% CI: 4.10–80.76; p = 1.36 × 10 ⁻ ⁴) for school-age children vs adults.

Next, we sought to evaluate the impact of matching narrower age ranges to better assess standard adult treatment in comparable age groups across endemic countries. Using age-matched data from Mozambique and South Africa, two studies comprising 2,790 adults aged 16–23 years revealed substantial variation in underdosing. In Mozambique, 8.42% of individuals in this age group received an insufficient dose, whereas in South Africa, up to 34% were treated with <30 mg/kg (Table 4). The random-effects meta-analysis for age-matched adults (16–23 years) estimated that 18.2% (95% CI: 3.8%–55.1%) of this population is likely to receive insufficient doses under the current WHO dose pole. A similar analysis was conducted for adults aged 15–49 years, using available data from two studies in Zimbabwe and Mozambique, which included a total of 7,791 individuals (Table 5). In this age group, the meta-analysis showed that 13.4% (95% CI: 10.6%-16.8%) received an insufficient dose (Fig 2 and S3 Dataset).

**Table 4:** Community-based treatment for adults (ages 16-23) using the WHO dose pole, categorized by dose (Insufficient, Acceptable, Optimal, and Excessive), from two studies in Mozambique and South Africa.

Community-based treatment (Matched)							
Country	Sample Size	Age range	Insufficient dose <30 mg/Kg	Acceptable dose 30-40 mg/Kg	Optimal dose 40-60 mg/Kg	Excessive dose >60 mg/Kg	Reference
Mozambique	641	16-23	54 (8.42%)	352 (55%)	232 (36.2%)	3 (0.46%)	Gazzinelli-Guimaraes et al., 2018 [22]
South Africa	2,149	16-23	744 (34.6%)	1,104 (51.4%)	299 (13.9%)	2 (0.1%)	Baan et al., 2016 [9]
**Total**	**2,790**	**16-23**	**798 (28.6% [±0.18%])**	**1,456** **(52.2% [±0.02%])**	**531** **(19% [±0.15%])**	**5 (0.2% [±0.002%])**	

**Table 5:** Community-based treatment for adults (ages15-49), categorized by dose (Insufficient, Acceptable, Optimal, and Excessive), using the WHO dose pole with matched ages (15-49) from two studies in Mozambique and Zimbabwe.

Community-based treatment (Matched)							
Country	Sample Size	Age range	Insufficient dose <30 mg/Kg	Acceptable dose 30-40 mg/Kg	Optimal dose 40-60 mg/Kg	Excessive dose >60 mg/Kg	Reference
Mozambique	2,177	15-49	258 (12%)	1,098 (50%)	809 (37.2%)	12 (0.6%)	Gazzinelli-Guimaraes et al., 2018 [22]
Zimbabwe	5,614	15-49	842 (15.0%)	2,970 (52.9%)	1,796 (32.0%)	6 (0.1%)	Palha de Souza et al., 2014 [23]
**Total**	**7,791**	**15-49**	**1,100 (14.1% [±0.02%])**	**4,068 (52.2% [±0.02%])**	**2,605 (33.45% [±0.04%])**	**18** **(0.23% [±0.003%])**	

## Discussion

As of 2022, over 260 million people in 50 countries required preventive chemotherapy for schistosomiasis; 21 of those countries were highly endemic areas that required both SAC and adults to receive treatment. Over 129 million adults were required to receive praziquantel during MDA, using the dose pole [2]. Overall, efforts to combat schistosomiasis include education, access to improved and safe water, vector control, and praziquantel as PC through MDA as one arm of efforts being deployed to lead to elimination. When praziquantel is given in an acceptable dose (defined by WHO guidelines) (30–60 mg/kg), a recent systematic review and meta-analysis of 52 clinical trials demonstrated that praziquantel produced a protection of 76% (95% CI from 67-83%) for human schistosomiasis, when compared to the placebo group [32].

In this systematic review, which includes 64,212 participants from 21 countries spanning the period from 1999 to September 2024, we demonstrate that the WHO dose pole achieves high proficiency in delivering sufficient doses of praziquantel to school-age children. However, its accuracy is questionable when applied to adults, with a substantial proportion receiving insufficient doses (<30mg/Kg).

Here, we showed that adults (aged 15–95) in Zimbabwe, South Africa, and Mozambique treated using the dose pole in a community-wide treatment setting had nearly 20% of the population underdosed (<30 mg/kg) in mass drug administration programs. As reported in studies in Mozambique [21] and South Africa [24], obesity (BMI > 25 kg/m2) actively drives the difference in administering a sufficient dose to adolescents and adults when using the dose pole, as the pole is a fixed measurement based on height and weight. While we do not know if obesity played a role in the SAC studies, it is a factor in adults, considering countries have experienced increased obesity rates since the development of the first dose pole. Respectively, over 30% of South African, 10% of Mozambican, and 14% of Zimbabwean adults are obese, with a body mass index (BMI) of 30 kg/m² or higher [33]. These numbers are expected to continue growing unless obesity is addressed in each country.

Two studies reported the use of the dose pole, where disparities in dosing and underdosing were observed across subgroups of sex and age [21,24]. The most significant difference is in the insufficient doses, with the age range of 16–23 being the most critical for treatment with the dose pole. Obesity is a growing issue in older children and adolescents than in primary school-aged children, and the rate of obesity in the adolescent population increases as they age, especially for women in the 16–23 age range. In adolescence, height and weight can vary and fluctuate; therefore, treatment with the current dose pole is most likely to deviate [16,24]. We saw this in our study; the most considerable amount of overdosing occurred in the 16–23 age range when compared to the larger age ranges of adults. We did not stratify the results by sex, but we would expect a similar number of males to experience this difference in dosing, especially in areas experiencing growing obesity. Though our review does not distinguish between females and males for dosing, women are more likely to be overweight when compared to men, particularly in low and middle-income countries [34]. Women have been targeted more for schistosomiasis intervention in recent years due to the occurrence of female genital schistosomiasis and its association with HIV [35]. In Mozambique, 18.3% of women were underdosed compared to 10.8% of men [21]. In South Africa, 3,157 females were investigated, and 35% were found to be overweight/obese, which contributed significantly to the 34% of underdosing among the adult women [24].

Using the current dose pole to treat adults raises this question: “Would adapting the dose pole increase the ability and efficiency to provide adults with acceptable or optimal dosing of praziquantel?” Due to the growing obesity rates worldwide [36], without further adaptation, using the dose pole for adults would likely lead to underdosing [21]. Palha de Sousa and colleagues proposed the addition of two intervals (3.5 and 4.5) to account for the differences in BMI, and administered additional tablets, as suggested by the dose pole with a 20% additional dose [23]. The addition comes into play when recognizing that praziquantel is safe when multiple doses are administered at a higher (60mg/kg) range to both children and adults [13]. Indeed, these two new pole labels led to the inclusion of more adults in the acceptable dose range; however, it is subjective and categorizes adults, based on observation, using singular images to estimate BMI to supply additional tablets, with the hope of correcting for those utilizing the dose pole that would receive improper doses. The subjectivity of this method stems from the lack of inclusion and acknowledgment of differing body types, as well as entrusting the decision to adjust the dosage to the person administering the dose. As reviewed by Strandgaard and colleagues [27], this method of correction for adult treatment has also been suggested by Alexander et al. [37], but was considered too complicated to be an accurate method for dosage determination.

Baan and collaborators’ assessment of the modified pole suggested one additional tablet for those observed as overweight (correcting for BMI), thereby decreasing the proportion of those who received insufficient doses from 6.3% (WHO dose pole) to 3.4% [24]. The modified dose pole was then evaluated in Mozambique, where 15% (n = 333/2,231) of the population were identified as overweight/obese (based on height-to-weight ratios). Besides the increase in the number of those receiving optimal dosages, for 21–55-year-old adult women, there was a significant reduction of underdosing from 21.3% to 15.5% [21]. This further suggests that adapting the WHO dose pole could be an effective method for delivering an accurate dose of praziquantel to adults. These papers recommend the development of a standardized universal dose pole to eliminate confusion and for overall ease of use across age ranges in endemic areas. However, it is important to mention that even with a modified dose pole, without corrections for BMI, millions of adults would still receive insufficient doses of praziquantel yearly. In addition, areas with high weight variability in each category of height would leave any alteration in the dose pole inappropriate.

The updated WHO guidelines and increased access to drugs through donation have generated hope for replicating the overall ease of treatment, as reflected in the use of the dose pole with SAC. However, the current dose pole does not proficiently treat adults, leading to repeated underdosing during MDA in communities that are the most vulnerable. The implication of repeated underdosing in adults has not been evaluated yet, but it could contribute to treatment failure and maintenance of the transmission in high-endemicity areas. While resistance has not been demonstrated in the field, it remains a concern, particularly with efforts to expand MDA and the potential for repeated underdosing in endemic areas. These studies, which represent community-wide treatment with adults, did not evaluate the potential for the selection of resistance.

In conclusion, this systematic review suggests that while the dose pole remains effective for SAC, its limitations for adults necessitate urgent adaptation or alternative dosing strategies to ensure equitable and effective treatment across all age groups. The solution for optimal dosing with praziquantel with the dose pole is complex and not “one size fits all”. Here, we showed that up to 34% of adults received insufficient doses in certain age groups, which could represent millions of underdoses yearly. The future use of the dose pole will require specific adaptations depending on the population being treated, as it is currently that height-based dosing misaligns with adult body metrics, particularly those who are overweight or obese, leading to significantly insufficient doses worldwide. Nevertheless, weight remains the most accurate method for delivering praziquantel to adult populations.

**Key points**

- In 2022, over 260 million people required preventive chemotherapy for schistosomiasis with praziquantel using the dose pole, including 129 million high-risk adults.
- The praziquantel dose pole achieves 96% accuracy in delivering optimal or acceptable doses to school-aged children.
- Up to 34% of adults in certain age groups received insufficient doses, particularly those who are overweight or obese.
- Height-based dosing misaligns with adult body metrics, leading to significant underdosing.
- Expanded mass drug administration to adults necessitates improved dosing strategies, including modifications to the dose pole or alternative weight-based dosing for effective adult treatment.

## Supporting information

S1 Fig Random-effects meta-analysis of optimal praziquantel dosing by treatment strategy and age range. Forest plots show the proportion of participants receiving optimal doses (40–60 mg/Kg) (red circles) for each study, estimated using the DerSimonian–Laird random-effects model. Panels represent: (A) school-based treatment of school-age children (SAC), (B) community-wide treatment including SAC and adults (0–95 years), (C) community-wide treatment of adults only (15–95 years), (D) age-matched adults (16–23 years), and (E) age-matched adults (15–49 years). The dotted vertical line indicates the pooled proportion for each dosing category, and the shaded area shows the corresponding 95% confidence interval (CI).
(S1_Fig.TIFF)

S1 Dataset All studies considered for screening based on the exclusion and inclusion criteria. (XLSX)

S2 Dataset Wilson Score proportion and confidence intervals for insufficient, acceptable/optimal and excessive doses and study treatment area. (XLS)

S3 Dataset Meta-analysis results by DerSimonian and Laird random-effects model (logit transformation). (XLSX)

S4 Dataset Wilson Score proportion and confidence intervals for optimal doses and study treatment area. (XLSX)

S5 Dataset Meta-analysis results by DerSimonian and Laird random-effects model (logit transformation) for optimal dosing across the studies. (XLSX)
