# Supplementary figures and images for: Evaluating the efficacy of the praziquantel dose pole for schistosomiasis treatment: A multi-country systematic review
Source: PLoS Negl Trop Dis. 2025 Oct 10;19(10):e0013587. doi: 10.1371/journal.pntd.0013587 (PMC12513654; doi:10.1371/journal.pntd.0013587)

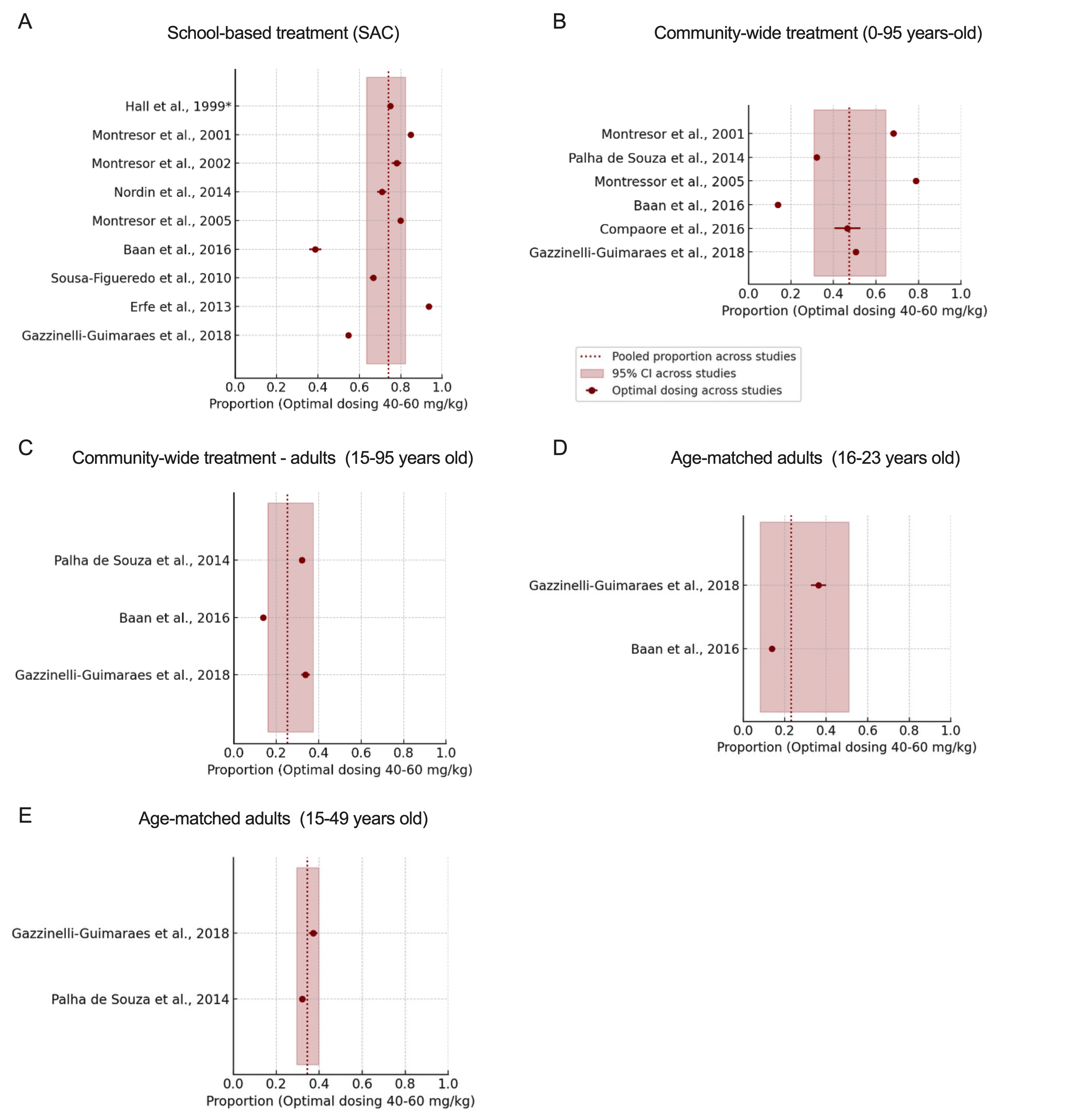

Supplement: S1 Fig — Forest plots show the proportion of participants receiving optimal doses (40–60 mg/Kg) (red circles) for each study, estimated using the DerSimonian–Laird random-effects model. Panels represent: (A) school-based treatment of school-age children (SAC), (B) community-wide treatment including SAC and adults (0–95 years), (C) community-wide treatment of adults only (15–95 years), (D) age-matched adults (16–23 years), and (E) age-matched adults (15–49 years). The dotted vertical line indicates the pooled proportion for each dosing category, and the shaded area shows the corresponding 95% confidence interval (CI). (S1_Fig.TIFF) [file pntd.0013587.s001.tiff]
